# Representation of pheromones, interspecific signals, and plant odors in higher olfactory centers; mapping physiologically identified antennal-lobe projection neurons in the male heliothine moth

**DOI:** 10.3389/fnsys.2014.00186

**Published:** 2014-10-09

**Authors:** Xin-Cheng Zhao, Pål Kvello, Bjarte B. Løfaldli, Siri C. Lillevoll, Hanna Mustaparta, Bente G. Berg

**Affiliations:** ^1^Department of Psychology, Norwegian University of Science and Technology (NTNU)Trondheim, Norway; ^2^Department of Biology/Neuroscience Unit, MTFS, Norwegian University of Science and Technology (NTNU)Trondheim, Norway

**Keywords:** standard brain atlas, antennal-lobe projection neurons, lateral horn, antennal-lobe glomeruli, macroglomerular complex (MGC)

## Abstract

The arrangement of anatomically separated systems for information about general and pheromone odorants is well documented at the initial levels of the olfactory pathway both in vertebrates and insects. In the primary olfactory center of the moth brain, for example, a few enlarged glomeruli situated dorsally, at the entrance of the antennal nerve, are devoted to information about female-produced substances whereas a set of more numerous ordinary glomeruli (OG) receives input about general odorants. Heliothine moths are particularly suitable for studying central chemosensory mechanisms not only because of their anatomically separated systems for plant odors and pheromones but also due to their use of female-produced substances in communication across the species. Thus, the male-specific system of heliothine moths includes two sub-arrangements, one ensuring attraction and mating behavior by carrying information about pheromones released by conspecifics, and the other inhibition of attraction via signal information emitted from heterospecifics. Based on previous tracing experiments, a general chemotopic organization of the male-specific glomeruli has been demonstrated in a number of heliothine species. As compared to the well explored organization of the moth antennal lobe (AL), demonstrating a non-overlapping representation of the biologically relevant stimuli, less is known about the neural arrangement residing at the following synaptic level, i.e., the mushroom body calyces and the lateral horn. In the study presented here, we have labeled physiologically characterized antennal-lobe projection neurons in males of the two heliothine species, *Heliothis virescens* and *Helicoverpa assulta*, for the purpose of mapping their target regions in the protocerebrum. In order to compare the representation of plant odors, pheromones, and interspecific signals in the higher brain regions of each species, we have created standard brain atlases and registered three-dimensional models of distinct uniglomerular projection neuron types into the relevant atlas.

## Introduction

Since the identification of the first pheromone, bombykol—produced by the *Bombyx mori* female (Butenandt et al., 1959), moths have been widely used for exploring how neural pathways encode olfactory information. As in other insects, the olfactory sensory neurons of the moth are situated on the antennae and project directly to the antennal lobe (AL), the primary olfactory center of the brain. Male moths possess a large number of sensory neurons specifically tuned to female-produced pheromones. These male-specific neurons, housed in long hair sensilla, target a small number of enlarged glomeruli situated dorsally in the AL at the entrance of the antennal nerve, the so-called macroglomerular complex (MGC; Boeckh and Boeckh, 1979; Matsumoto and Hildebrand, 1981; Kanzaki and Shibuya, 1986). The sensory neurons tuned to general odors, present in both sexes and situated in short hair sensilla, project to the numerous ordinary glomeruli (OG). After being processed in the AL, the olfactory information is carried by projection neurons targeting mainly two higher integration regions in the protocerebrum, the calyces of the mushroom bodies and the lateral horn (here, referred to as the region of the lateral protocerebrum being targeted by the antennal-lobe projection neurons, as suggested by Ito et al., 2014; reviewed by Galizia and Rössler, 2010). Of several output tracts, the medial antennal-lobe tract (mALT; Ito et al., 2014), is the most prominent one (Homberg et al., 1988; Rø et al., 2007). Due to the number and diameter of axons passing in this tract it appears as the thickest in mass-stained preparations (Homberg et al., 1988). The mALT consists mainly of uniglomerular projection neurons targeting the calyces before continuing to the lateral horn.

Among the moth species most intensively studied is a handful of the monophyletic subfamily, heliothinae. By tracing functionally characterized receptor neurons and antennal-lobe projection neurons, a general chemotopic organization of the male-specific MGC has been demonstrated in a number of geographically isolated and sympatric heliothine species including the American tobacco budworm moth, *Heliothis virescens* (Christensen et al., 1995; Hansson et al., 1995; Berg et al., 1998; Vickers et al., 1998; Vickers and Christensen, 2003), *Heliothis subflexa* (Baker et al., 2004), *Helicoverpa zea* (Christensen et al., 1991; Cossé et al., 1998; Vickers et al., 1998; Lee et al., 2006), and *Helicoverpa assulta* (Berg et al., 2005; Zhao and Berg, 2010). Especially intriguing, is the use of chemical signals in communication both within and across the species in this group. Thus, the male-specific system includes two sub-arrangements, one ensuring attraction and mating behavior by carrying information about pheromones released by conspecifics, and the other preventing mating mistakes via signal information emitted from heterospecifics (Mustaparta, 1996). For example, males of *H. vires*c*ens* possess two types of pheromone sensory neurons tuned to *cis*-11-hexadecenal (Z11-16:AL) and *cis*-9-tetrdecenal (Z9-14:AL), respectively, whereas two additional neuron populations are tuned to substances produced by females of sympatric species, one to *cis*-11-hexadecenyl acetate (Z11-16:AC) and the other to *cis*-11- hexadecenol (Z11-16:OH; Almaas et al., 1991; Berg et al., 1995, 1998; Hansson et al., 1995). In males of the Oriental tobacco moth, *H. assulta*, two types of pheromone sensory neurons are tuned to *cis*-9-hexadecenal (Z9-16:AL) and Z11-16:AL, respectively, whereas a population of Z9-14:AL-responding neurons carries interspecific information (Berg and Mustaparta, 1995; Berg et al., 2005).

As compared to the well explored organization of the moth AL, demonstrating a non-overlapping representation of the biologically relevant stimuli, less is known about the neural arrangements residing at the following synaptic level, i.e., the calyces and the lateral horn. In two insect species, however, the silk moth, *Bombyx mori*, and the fruit fly, *Drosophila melanogaster*, specific areas in the protocerebrum receiving projection terminals of pheromone and general odor antennal-lobe output neurons, respectively, have been reported. Thus in, *B. mori*, uni-glomerular MGC-neurons project to a region of the lateral horn previously referred to as “the delta area of the inferior lateral protocerebrum” whereas the neurons linked to OG project to a more posterior-lateral area of the lateral horn (Kanzaki et al., 2003; Seki et al., 2005). Furthermore, the male-specific MGC-projections are subdivided; those originating in the toroid, carrying information about the major pheromone component, bombykol, target a more medially located region than the neurons tuned to the second component, bombykal (Kanzaki et al., 2003; Seki et al., 2005). A somewhat different kind of organization was recently reported for the calyces by the major pheromone component being represented in a region different from those of plant-odors and the minor pheromone component which display a large degree of overlap (Namiki et al., 2013). In studies on the fruit fly, *Drosophila melanogaster*, combining single-cell labeling and image registration, partly corresponding findings have been reported by spatially segregated areas for fruit and pheromone signals in the higher olfactory centers (Jefferis et al., 2007). In these studies, which included projections not only in the mALT but also in the mediolateral ALT, fruit odors were found to be represented mainly in the posterior-dorsal region of the lateral horn and the pheromone more anterior-ventrally. With respect to the innervation pattern in the mushroom body calyces of the fly, inputs from the different antennal-lobe glomeruli are reported to have distinct representations (Jefferis et al., 2007; Lin et al., 2007). Furthermore, in a recent study using photoactivatable fluorescent proteins in combination with electrophysiology and optical imaging, Ruta et al. (2010) have reported about male-specific lateral-horn neurons in a dimorphic pheromone pathway in the fruit fly.

In the study presented here, we have labeled physiologically characterized antennal-lobe projection neurons in males of the two heliothine species, *H. virescens* and *H. assulta*, for the purpose of mapping their target regions in the higher brain centers. The advantage of using heliothine species—in addition to their two arrangements of glomeruli being related to insect-produced compounds and plant odors, respectively—is that their MGCs consist of easily identifiable glomeruli being linked to distinct behavioral responses. In order to compare the representation of the particular glomeruli in the higher brain centers of each species, we have created standard atlases of the central brain for the above mentioned heliothine moths and registered three-dimensional models of uniglomerular projection neurons into the relevant brain atlas. By selecting two related species using different components as the major pheromone constituent we could determine whether this particular distinction is reflected in the projection pattern as well.

## Materials and methods

### Insects and preparation

Pupae of *H. virescens* and *H. assulta* were imported from laboratory cultures (*H. virescens* from Syngenta, Basel, Switzerland; and *H.assulta* from Henan University of Science and Technology, Henan, China). The pupae were separated based on sex and kept in climate chambers on phase-shifted photoperiod LD 14–10 h at 22°C. The adults were fed a 5% sucrose solution. Adult males were used for the experiments 2–5 days after ecdysis. The moth was restrained inside a plastic tube with the head exposed and then immobilized with dental wax (Kerr Corporation, Romulus, MI). The brain was exposed by opening the head capsule and supplied with Ringer’s solution (in mM: 150 NaCl, 3 CaCl2, 3 KCl, 25 Sucrose, and 10 N-tris (hydroxymethyl)-methyl-2-amino-ethanesulfonic acid, pH 6.9).

### Intracellular recording and staining

The intracellular recordings from the antennal-lobe neurons were carried out as previously described (Zhao and Berg, 2010). Recording electrodes were made by pulling glass capillaries (Borosilicate glass capillaries, Hilgenberg GmbH, Germany; OD: 1 mm, ID: 0.75 mm) on a horizontal puller (P97, Sutter Instruments, Novarto, CA). The tip was filled with a fluorescent dye (4% tetramethylrhodamine dextran with biotin, Micro-Ruby, Molecular Probes; Invitrogen, Eugene, OR; in 0.2 M K^+^-acetate) and the glass capillary back-filled with 0.2 M K^+^-acetate. A chloridized silver wire inserted into the eye served as the indifferent electrode. The recording electrode, which had a resistance of 150–400 MΩ, was lowered carefully into the dorsolateral region of the AL. Neuronal spike activity was amplified (AxoClamp 2B, Axon Instruments, Union, CA) and monitored continuously by oscilloscope and loudspeaker. Spike2 6.02 software (Cambridge Electronic Design, Cambridge, England) was used as acquisition software.

After physiological characterization of responses to the test stimuli, the neurons were iontophoretically stained by applying 2–5 nA depolarizing current pulses with 200 ms duration at 1 Hz for about 2–10 min via the glass capillary electrode. In order to allow neuronal transportation of the dye, the preparation was kept for 2 h at room temperature. The brain was then dissected from the head capsule. After being fixed in 4% paraformaldehyde for 1 h at room temperature, the brain was rinsed with a phosphate-buffered saline (PBS; in mM: 684 NaCl, 13 KCl, 50.7 Na2HPO4, 5 KH2PO4, pH 7.4). The staining was then intensified by incubating the brain in fluorescent conjugate streptavidin-Cy3 (Jackson Immunoresearch, West Grove; PA, diluted 1:200 in PBS), which binds to biotin, for 2 h. Incubation was followed by rinsing with PBS and dehydration in an ascending ethanol series (50%, 70%, 90%, 96%, 2 × 100%; 10 min each). Finally, the brain was cleared and mounted in methylsalicylate.

### Odor and air puff stimulation

The odor delivery system for the intracellular recordings consisted of two glass cartridges (ID 0.4 cm) placed side by side, both pointing toward the antenna at a distance of 2 cm. One replaceable cartridge contained a piece of filter paper onto which a particular odor stimulus was applied. The other cartridge contained a clean filter paper. An air flow (500 ml/min) led through the odorless cartridge was continuously blown over the antenna. During each stimulus period, which lasted for 400 ms, the air flow was switched by a valve system from the odorless to the odor-bearing cartridge. As olfactory stimuli we used the two-component pheromone blends of the two species, i.e., Z11-16:AL and Z9-14:AL in the ratio 94:6 for *H. virescens* and Z11-16:AL and Z9-16:AL in the ratio 5:95 for *H. assulta* (Pheromone chemicals, Plant Research International, Pherobank, Wageningen, Netherlands). Also, we tested the single constituents of the two pheromone blends, plus the interspecific signals, Z11-16:AC and Z11-16:OH on *H. virescens* and Z9-14:AL on *H. assulta*. In addition, the plant oil ylang-ylang was used (Dragoco, Totowa, NJ). The odor compounds, diluted in hexane, were applied onto a small filter-paper. The hexane was allowed to evaporate in a weak stream of pure nitrogen before the filter-paper was wrapped up and placed in the cartridge. All stimuli were prepared so that the filter-paper contained 10 ng of the single insect-produced substances and the binary pheromone blend, and 100 μg of the plant oil. A cartridge containing a pure filter paper was used as control. The odor stimuli were regularly renewed during the experimental period.

### Mass stainings

Mass staining experiments were performed on 10 *H. virescens* males. The MGC region and the region containing OG in the AL were perforated with fine needles. Crystals of two fluorescent dyes were then applied to the AL by means of the fine tip of a micro needle, micro-ruby into the MGC and micro-emerald (Molecular Probes)/Alexa 488 (Life Sciences) into the OG. The brain was subsequently supplied with Ringer’s solution and kept for 2 h at room temperature for transportation of the dyes. The following procedure including dissection, fixation, dehydration, and mounting in methylsalicylate was as described above.

### Immunocytochemistry

To create standard brain models, immunostaining with an antibody marking synaptic regions, anti SYNORF1, was performed. The anti SYNORF1 (Developmental Studies Hybridoma Bank, University of Iowa) was raised against fusion proteins composed of glutathione-S-transferase and the *Drosophila* SYN1 protein (SYNORF1; Klagges et al., 1996). The specificity of this antibody has been described by Klagges et al. (1996), and it is reported to detect synaptic neuropil in various insect species, heliothine moths included (Berg et al., 2002; Kvello et al., 2009). The brain was dissected from the head capsule and fixed in 4% paraformaldehyde for 1 h at room temperature before being rinsed in PBS 4 × 15 min. Subsequently, the brain was preincubated in 5% normal goat serum (NGS, Sigma, St. Louis, MO, USA) in PBSX (PBS containing 0.5% Triton-X 100, pH 7.4). The preparation was then incubated for 2 days at 4°C in the primary antibody, anti SYNORF1 (dilution 1:10 in PBSX containing 5% NGS). After rinsing in PBS 6 × 20 min at room’s temperature, the brain was incubated with Cy2-conjugated anti-mouse secondary antibody (Invitrogen, Eugene, OR; dilution 1:500 in PBSX) for 2 days at 4°C. Finally, we rinsed, dehydrated, cleared, and mounted the brain in methylsalicylate.

In order to identify glomeruli and neuropil regions in brains with labeled neurons, immunostaining with SYNORF1 was performed. After analyzing the iontophoretically stained neuron by confocal laser scanning microscopy, the brain was rehydrated through a decreased ethanol series (10 min each) and rinsed in PBS. Then the brain was immunostained with anti SYNORF1, as described above.

### Confocal image acquisition

The serial optical images were obtained by using a confocal laser scanning microscope (LSM 510, META Zeiss, Jena, Germany) with a 10× (C-Achroplan 10×/0.45 W), 20× (Plan-Neofluar 20×/0.5), and 40× objective (C-Achroplan 40×/0.8 W). The intracellular and mass staining, obtained from the fluorescence of rhodamine/Cy3 (Exmax 550 nm, Emmax 570 nm), was excited by the 543-nm line of a HeNe1 laser whereas the mass staining obtained from micro-emerald/Alexa 488, plus the immunostaining obtained from the Cy2 (Exmax 490 nm, Emmax 508 nm), were excited by the 488-nm line of an Argon laser. The distance between each section was 3–6 μm for the 10× objective and 2 μm for the 20× and 40× objectives. The pinhole size was 1 and the resolution 1024 × 1024 pixels. Optical sections from the confocal stacks were reconstructed by means of the LSM 510 projection tool.

### Generation of standard brain models

The relevant brain structures were manually reconstructed from the confocal slices by using the segmentation editor of the software AMIRA 4.1 (Visage Imaging, Fürth, Germany). Each brain structure was outlined based on its gray value according to the background so that a polygonal surface model could be created. Ten brains of *H. virescens* and five of *H. assulta* were used to create the standard brain models. For standardization, the iterative shape averaging (ISA) protocol was used, as previously described in Brandt et al. (2005) and Kvello et al. (2009). For each species, one brain preparation was selected as a template. The label images of the other brain preparations were subsequently aligned to the label images of the template brain using affine registration. This process repositions, rotates, and scales the label images of the brain preparations to match the template brain. All labeled images were then used to generate an average brain. Subsequently, the affine registered brains and the template brain were elastically registered to the average brain. This procedure compensates for local differences in the label images of the brain preparations and the average brain. This was followed by a second averaging procedure producing a second average brain. The elastic registration and the averaging procedure were repeated three times. The average brain generated after the third elastic registration was used as a standard brain model.

### Reconstruction and registration of neurons into the standard brain model

The neuron and the surrounding brain neuropils were manually reconstructed in subsequent confocal slices by means of the visualization software AMIRA 4.1; the neuron was reconstructed by using the skeleton module of the software (Evers et al., 2004; Schmitt et al., 2004), and the brain structures by using the segmentation editor, as described above. Thus, the neuron was traced so that a surface model built by cylinders of particular lengths and thicknesses was created. The neuronal structures and the neuropil regions were reconstructed from the same confocal image stacks. The z-axis dimension of the neuron and brain structures was multiplied by a factor of 1.3 for the water lens objective and 1.6 for the dry lens, to compensate for the refraction indexes.

Registration of the neurons into the standard brain model followed the same procedure as described in Brandt et al. (2005), Kvello et al. (2009), and Løfaldli et al. (2010). The label images of the brain preparations with stained neurons were affine and elastically registered to the corresponding label images of the standard brain. The resulting transformation parameters for the brain structures were subsequently applied to the reconstructed neurons. The results were then carefully evaluated by comparing the confocal images with the obtained model.

### Data analyses and image processing

The images were adjusted in Photoshop CS3 by means of the auto-contrast tool before the final figures were edited in Adobe Illustrator CS3. The orientation of all brain structures is indicated relative to the body axis of the insect, as in Homberg et al. (1988).

## Results

### Reconstruction of the brain reference frameworks and drafting of standard models

**Figure 1:**
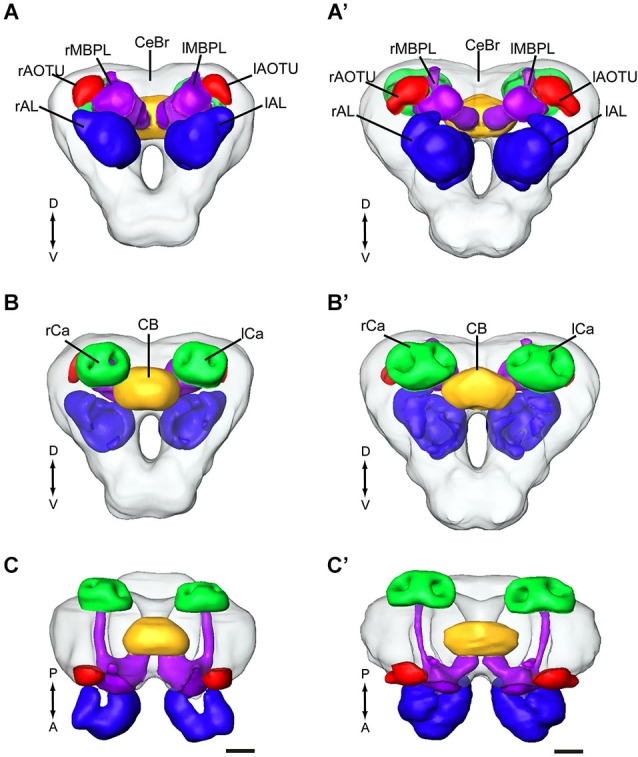
**Average standard brain models of the two heliothine species, *Helicoverpa assulta* and *Heliothis virescens* (the optic lobes are not included)**. **(A–C)** *H. assulta*. **(A’–C’)** *H. virescens*. **(A,A’)** Anterior view. **(B,B’)** Posterior view. **(C,C’)** Dorsal view. CB, central body; lAL, left antennal lobe; lAOTU, left anterior optic tubercle; lCa, left calyces; lMBPL, left mushroom body peduncle and lobes; CeBr, central brain; rAL, right antennal lobe; rAOTU, right anterior optic tubercle; rCa, right calyces; rMBPL, right mushroom body peduncle and lobes. A, anterior; D, dorsal; P, posterior; V, Ventral. Scale bars, 100 μm.

In order to make standard models of the relevant brain regions of the two heliothine species, *H. assulta* and *H. virescens*, we chose the most successfully stained preparations for constructing images of particular neuropil structures. The brain model of *H. assulta* was thus created by selecting five of totally nine stained preparations whereas that of *H. virescens* was made by selecting 10 of totally 17 stained preparations. The selection of reconstructed brain structures comprised the central brain (for terminology, see Ito et al., 2014), the AL, the anterior optic tubercle, the central body, the mushroom body lobes, and the calyces. According to the aim of the present study, which was to map the target regions of antennal-lobe projection neurons, the optic lobes were left out. The standard brain model for each of the two species is shown in a frontal, posterior, and dorsal view in Figure 1. To confirm that these models indeed were an average, we compared the shape variation of all brain preparations to each other, as well as to the average, using the surface distance tool in Amira 4.1, like in Kvello et al. (2009). These calculations revealed that among all the reconstructed brains, the standard model was the one being most similar to the others (data not shown).

### Mass staining of output neurons originating in the MGC and the ordinary glomeruli, respectively

**Figure 2:**
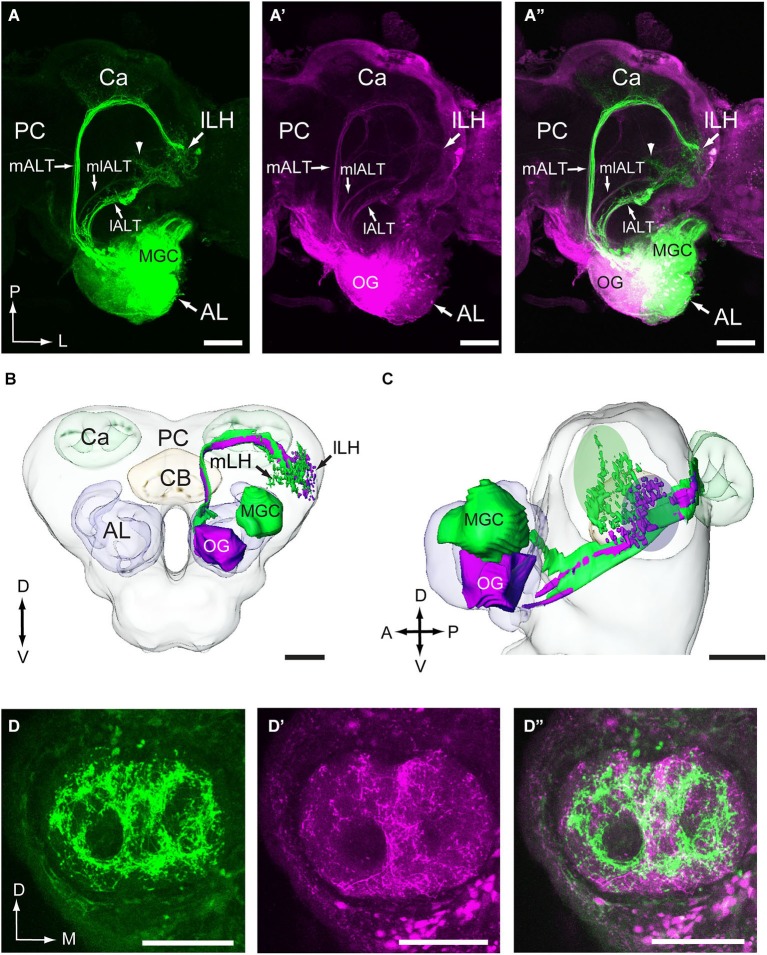
**Visualization of the main antennocerebral pathways obtained by selective staining of projection neurons linked to the macroglomerular complex (MGC) and ordinary glomeruli (OG)**. **(A–A”)** The three main antennal-lobe tracts (ALTs), the medial ALT (mALT), the medio-lateral ALT (mlALT), and the lateral ALT (lALT) are indicated by arrowheads. The image to the left shows the pathways of male-specific neurons, obtained by applying one particular dye into the MGC region (indicated by green). The image in the middle shows the pathways of neurons linked to the OG, obtained by applying another dye into the more ventral region of the (AL; indicated by magenta). The image to the right shows the overlay of the two former images. **(B,C)** Reconstructions of the male-specific neurons (green) and the plant odor neurons (magenta) projecting in the mALT showing that the two neuron categories target slightly different regions in the lateral horn. The ovals in green and magenta in **(C)** indicate the target areas of the medial-tract neurons originating from the MGC and the OGs, respectively. The reconstruction was made from one double-labeled preparation, i.e., the one presented in **(A)**. **(D–D”)** Confocal images of another double-labeled preparation showing distinctly different staining patterns of male-specific neurons (green) vs. plant odor neurons (magenta) in the calyces (Ca). The two categories show only some extent of overlap. lLH, lateral part of the lateral horn; mLH, medial part of the lateral horn; PC, protocerebrum. A, anterior; D, dorsal; L, lateral; M, medial; P, posterior; V, ventral. Scale bars, 100 μm.

The results from the mass staining experiments showed that the neuron categories linked to the male-specific and the OG, respectively, are present in all three main ALTs (Figures 2A–A”). In this study, we have reconstructed the fibers passing in the prominent medial ALT. As demonstrated in Figures 2B,C, the medial-tract neurons originating in the MGC target an area in the lateral horn, located slightly more medially and anteriorly to that innervated by the neurons originating in the OG, the so-called delta area of the moth brain. Also in the calyces, the two neuron categories showed different projection patterns. Thus, the collaterals of the MGC-neurons targeted mainly the inner part of the calyces, forming a ring-shaped structure within each cup (Figure 2D). The strong staining was probably due to the presence of numerous projections terminating in overlapping areas. The neurons originating from the OG, on the other hand, displayed a pattern of more evenly distributed terminal processes covering a larger region (Figure 2D’). As shown in the overlay in Figure 2D”, there is only minimal overlap between the terminals of the two neuron categories in the calyces. Both the collaterals of the plant odor and the male specific neurons extended from the medial ALT at right angles.

### Staining and reconstruction of uni-glomerular projection neurons

Among all stained antennal-lobe projection neurons achieved from each species during the intracellular recordings, we selected only those passing in the mALT for reconstruction and registration into the relevant standard atlas. The neurons incorporated in the study presented here are therefore of the uni-glomerular type sending off projections into the calyces before terminating in the lateral horn (Figures 2B,C). In *H. assulta*, a total number of 11 successfully stained uni-glomerular projection neurons originating from 10 brain preparations were reconstructed and registered into the reference framework, and seven neurons from three brains of *H. virescens* were included into the framework of this species (Some of the neurons obtained from *H. assulta* have been published previously, in Zhao and Berg (2010). The former study did not compare the target regions of the particular neurons, however).

### Visualization of individual antennal-lobe projection neurons in the standard atlas of *H. assulta*

**Figure 3:**
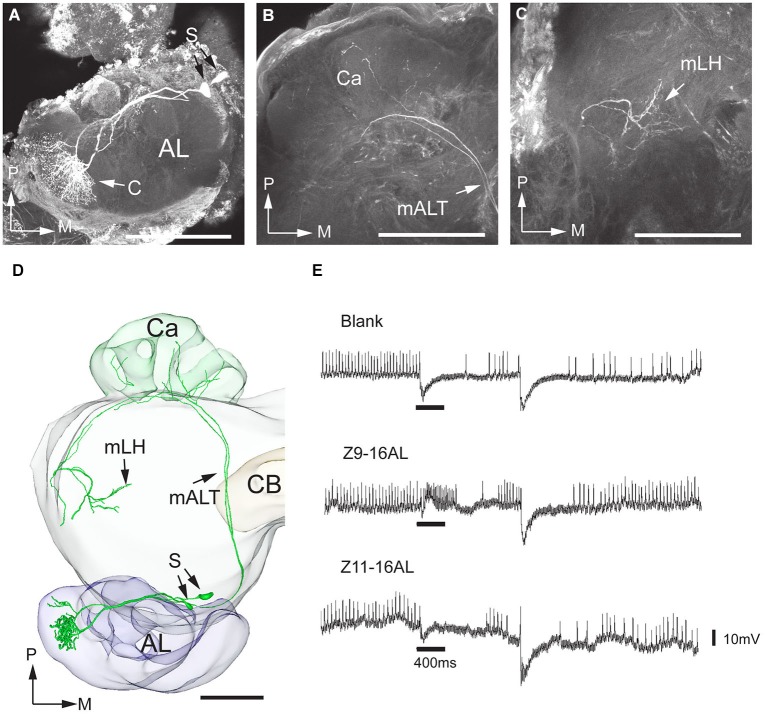
**Morphology and physiology of projection neurons linked to the cumulus (C) of the MGC in an *H. assulta* male**. **(A)** Two projection neurons can be seen, both having dendrites in the cumulus. **(B)** Projections in the calyces (Ca). **(C)** Terminal processes in the medial part of the lateral horn (mLH). **(D)** 3D-reconstruction of the two projection neurons and surrounding brain structures. **(E)** The electrophysiological recording shows an excitatory response to the principal pheromone component, Z9-16:AL (The deflections of the membrane potential which occur at the onset of the odor puff and the re-onset of the continuous air stream are probably due to a mechanical artifact). AL, antennal lobe; medial antennal-lobe tract mALT; S, somata; M, medial; P, posterior. Scale bars, 100 μm.

**Figure 4:**
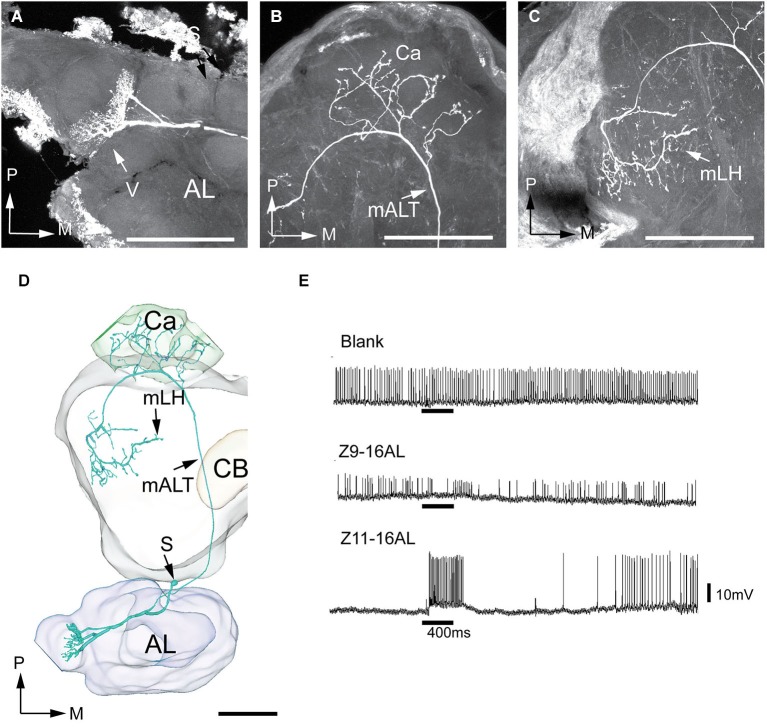
**Morphology and physiology of one projection neuron originating in the ventral glomerulus of the MGC in an *H. assulta* male**. **(A)** The dendrites in the ventral MGC glomerulus. **(B)** Axon branches in the calyces (Ca). **(C)** Axon terminals in the medial part of the lateral horn (mLH). **(D)** 3D-reconstruction of the projection neuron and the surrounding brain structures. **(E)** Electrophysiological data showing an excitatory response to the second pheromone component, Z11-16:AL. AL, antennal lobe; CB, central body; mALT, medial antenno-protocerebrum tract; mLH, medial part of the lateral horn; S, soma; V, ventral glomerulus; M, medial; P, posterior. Scale bars, 100 μm. (Adapted from Zhao and Berg, 2010).

**Figure 5:**
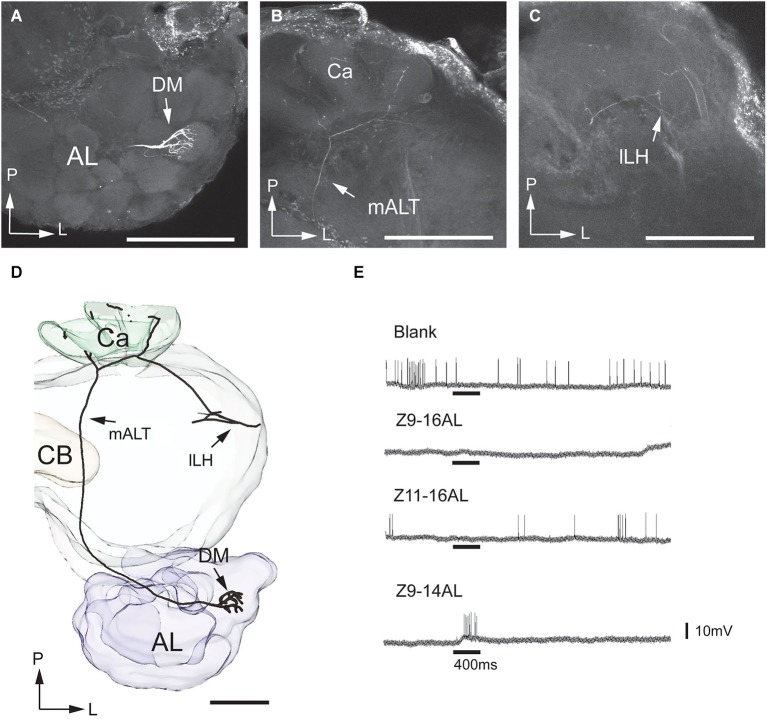
**Morphology and physiology of one projection neuron linked to the dorsomedial glomerulus of the MGC in an *H. assulta* male**. **(A)** The uniglomerular neuron with arborizations in the dorsomedial MGC glomerulus. **(B)** Processes in the calyces (Ca). **(C)** Terminals in the lateral part of the lateral horn (lLH). **(D)** 3D-reconstruction of the projection neuron and surrounding brain structures. **(E)** Electrophysiological data showing an excitatory response to the interspecific signal, Z9-14AL. AL, antennal lobe; CB, central body; DM, dorsomedial glomerulus; mALT, medial antennal-lobe tract. L, lateral; P, posterior. Scale bars, 100 μm.

**Figure 6:**
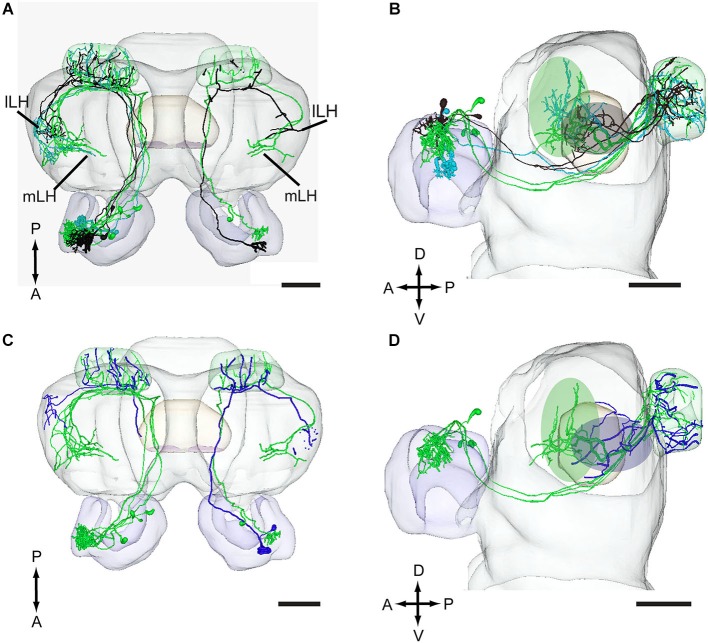
**Projection neurons originating in the MGC and OG registered into the standard brain model of *H. assulta*. (A,B)** Projection neurons arising from each of the three MGC units, the cumulus (green), the ventral unit (turquoise), and the dorsomedial unit (black), in dorsal view **(A)** and lateral view **(B)**. The green and gray ovals indicate target areas of projection neurons for the pheromone compounds and the interspecific signal, respectively. **(C,D)** Projection neurons originating in the MGC units and the OG in a dorsal **(C)** and a lateral **(D)** view. The green and blue ovals indicate target areas of projection neurons for pheromones and plant odors, respectively. lLH, lateral part of lateral horn; mLH, medial part of the lateral horn. A, anterior; D, dorsal; L, lateral; P, posterior. Scale bars, 100 μm.

Out of the 11 successfully stained projection neurons achieved in *H. assulta*, nine were connected to the MGC and two to OG. The former category, the male-specific neurons, included three sub-types, each originating in one of the three MGC-units—two sub-types responding to the pheromone compounds and the third to the interspecific signal. One pheromone sub-type was linked to the largest MGC-unit, i.e., the cumulus. An example is given in Figure 3 showing two simultaneously stained projection neurons, both arborizing in the cumulus. The recording underlying this staining revealed an excitatory response to the major pheromone component, Z9-16:AL (Figure 3E). The two neurons targeted overlapping regions in each of the higher brain centers (Figure 3D). The innervated area in the lateral horn was located in the medial part. The projections of the second type of pheromone-neuron, responding to Z11-16:AL and originating in the ventral MGC-unit, targeted the similar region of the lateral horn after giving off projections to the calyces (Figure 4). The third neuron category, being activated by the interspecific signal, Z9-14:AL, and arborizing in the dorso-medial MGC-unit, showed a somewhat different projection pattern by covering a considerably smaller area of the lateral horn (Figure 5). When registering all the reconstructed neurons of the male-specific category into the standard atlas, including totally four Z9-16:AL-responding neurons linked to the cumulus, one Z11-16:AL-responding neuron linked to the ventral unit, and four Z9-14:AL-responding neurons linked to the dorso-medial unit, it appeared that the pheromone information is represented in a distinct region partly separated from that of the interspecific signal in the lateral horn (Figures 6A,B). Thus, the pheromone neurons targeted an overlapping region being located slightly more medially, dorsally, and anteriorly than that innervated by the category responding to the non-pheromonal substance, Z9-14:AL. Also, the target area of the pheromone neurons was larger than that of the non-pheromonal type. As concerns the staining pattern in the calyces, the registration of reconstructed neurons from different individuals into the standard atlas did not show any distinctions between the various neuron types.

In addition to the male-specific neurons, reconstructed models of two stained projection neurons arborizing in OG were incorporated in the standard atlas. Both neurons responded to the plant odor mixture ylang-ylang (data not shown). As compared to the target region of the pheromone neurons, the projections of the plant odor-neurons covered a smaller area located more laterally and posteriorly in the lateral horn (Figures 6C,D).

### Visualization of individual antennal-lobe projection neurons in the standard atlas of *H. virescens*

**Figure 7:**
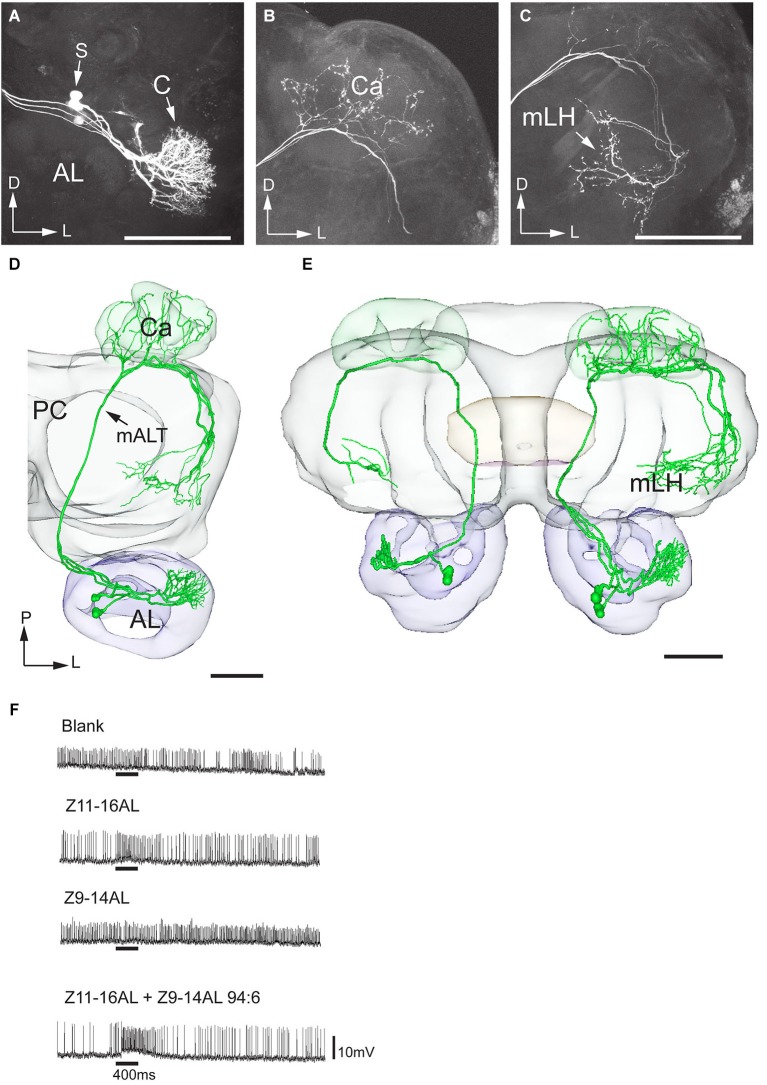
**Morphology and physiology of projection neurons linked to the cumulus (C) of the MGC in an *H. virescens* male**. **(A)** Three projection neurons, all having dendritic arborizations in the cumulus. **(B)** Projections in the calyces (Ca). **(C)** Axon terminals in the medial part of the lateral horn (mLH). **(D)** 3D-reconstruction of the three projection neurons and surrounding brain structures. **(E)** Five projection neurons arborizing in the cumulus have been registered into the standard brain model demonstrating their similar target region in the medial part of the lateral horn (mLH). **(F)** Electrophysiological data from one of the projection neurons presented in **(A–D)** showing excitatory responses to the major pheromone compound, Z11-16AL, and to the blend. AL, antennal lobe; C, cumulus; CB central body; mALT medial antennal-lobe tract; PC, protocerebrum; S, somata; D, dorsal; L, lateral; P, posterior. Scale bars, 100 μm.

Of the seven successfully stained antennal-lobe projection neurons achieved in *H. virescens*, five belonged to the male-specific category arborizing in the largest MGC-unit, the cumulus, and two in OG. An example of the MGC-category is presented in Figure 7. Here, three output neurons originating in the cumulus were simultaneously stained. The three neurons appeared tightly joined not only in the AL, but also in the medial tract and in the two termination regions. These neurons were therefore reconstructed together. In the calyces, the staining pattern included characteristic condensed structures assumingly made up by accumulations of terminals from each individual neuron (Figure 7B). Also, the axon terminals of the three neurons projected closely together and seemed to share common target areas in the lateral horn (Figure 7C). Here, they covered a relatively large region located in the medial part of the present area. The recording underlying the current labeling showed a response to the major pheromone component, Z11-16:AL and to the species-specific pheromone mixture (Figure 7F). The two remaining neurons linked to the cumulus, originating from another individual, showed a similar morphology by projecting in the same region of the lateral horn. Figure 7E, shows all five cumulus-neurons registered into the standard brain framework.

**Figure 8:**
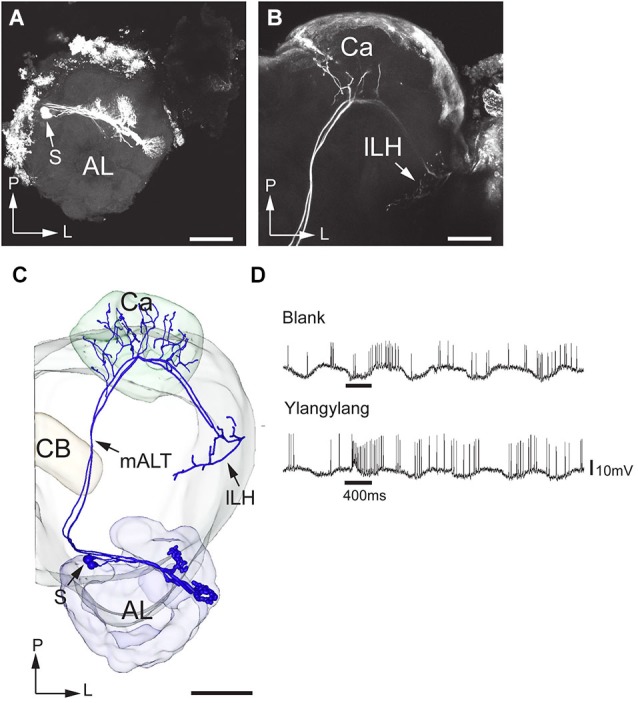
**Morphology and physiology of projection neurons connected to OG in an *H. virescens* male**. **(A)** Two projection neurons having dendrites in two OGs. **(B)** Axon projections in the calyces (Ca) and the lateral part of the lateral horn (lLH). **(C)** 3D-reconstruction of the projection neurons and surrounding brain structures. **(D)** Electrophysiological data (from one of the two stained neurons) showing excitatory responses to ylang-ylang. AL, antennal lobe; CB, central body; mALT, medial antennalo-lobe tract; S, somata; L, lateral; P, posterior. Scale bars, 100 μm.

**Figure 9:**
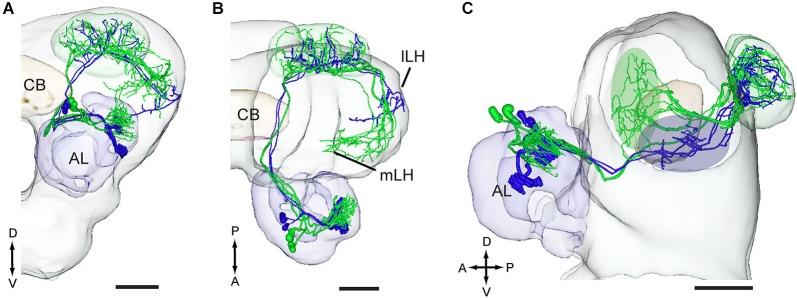
**Three-D models of pheromone neurons (green) and plant odor neurons (blue) registered into the standard brain model of the *H. virescens* male**. **(A)** Frontal orientation. **(B)** Dorsal orientation. **(C)** Sagittal orientation. The green and blue ovals indicate the different target regions of the two neuron categories in the lateral horn. lLH, lateral part of the lateral horn; mLH, medial part of the lateral horn. A, anterior; D, dorsal; L, lateral; P, posterior. Scale bars, 100 μm.

Two projection neurons arborizing in OG, stained simultaneously from the same brain, originated from one recording that showed an excitatory response to the plant odor mixture ylang-ylang (Figure 8). By placing all neuron models into the reference framework, including those linked to the MGC and the OG, respectively, the partly different target regions of the two neuron categories in the lateral horn appeared. Similarly to the findings obtained from *H. assulta*, the male-specific neurons in *H. virescens* projected to an area located more medially and anteriorly than those linked to the OG (Figure 9).

## Discussion

One purpose of making standard brain atlases of insect species is to obtain a common framework for integrating morphologically identified neurons from different brain preparations of a species. The ISA procedure, especially suitable for this kind of integration, has been used for creating standard brains of several insect species (Brandt et al., 2005; Kurylas et al., 2008; el Jundi et al., 2009). In particular, the usefulness of such a tool has been demonstrated by the standard brain atlas of *H. virescens* females including integrated gustatory and olfactory neurons (Kvello et al., 2009; Løfaldli et al., 2010). In order to compare the position of olfactory neurons carrying information about pheromones vs. plant odors, models of the male brain were required both in *H. virescens* and *H. assulta*. Using the ISA procedure, we here present standard atlases of the central brains of the two heliothine species serving as a framework for comparing the final target regions of stained projection neurons obtained from different individuals. The presented neurons are all included in the category of antennal-lobe projection neurons running in the mALT. The main outcome of the investigation is that different odor signals eliciting particular behavioral responses—including plant odors, pheromones, and interspecific signals—are represented in partly distinct subfields of the lateral horn. Thus, a general pattern of pheromone and plant odor neurons projecting to dissimilar regions in the lateral horn was found in the present study. In addition, the data from *H. assulta*, including two functionally different male-specific projection neuron categories, one pheromone type responsible for the male sexual behavior and one non-pheromonal type underlying inhibition of the sexual attraction, displayed partly different projection patterns in the lateral horn.

### Representation of plant odors vs. pheromones in the lateral protocerebrum

The arrangement of anatomically separated systems for information about general and pheromone odorants is well documented at the initial levels of the olfactory pathway both in vertebrates and insects (Hildebrand and Shepherd, 1997). Whether the two information pathways are kept separated in higher olfactory areas of the brain was one of the questions in the present study. Indeed, the results—including individually stained neurons and neurons labeled via mass staining—showed a separation of the two neuron categories in the lateral horn. In both species studied, *H. virescens* and *H. assulta*, the plant odor responding neurons with dendrites in OG projected in a region situated laterally and posteriorly to that targeted by the pheromone responding neurons, indicating the general validity of the particular projection pattern in heliothine moths. A similar kind of spatial separation has previously been reported in other moth species. Thus, in the silk moth, *B. mori*, toroid neurons tuned to the pheromone, bombykol, target the so-called delta region of the lateral horn whereas neurons from the OG project to an area located more posteriorly and laterally (Seki et al., 2005). Corresponding innervation patterns have been found for cobalt-stained projection neurons originating from the MGC vs. the OG in the sphinx moth, *M. sexta*, as well (Homberg et al., 1988). Also, in the fruit fly, *Drosophila melanogaster*, pheromones and general odorants are associated with different regions of the lateral horn—fruit odors with the posterior-dorsal part and pheromones with the anterior-ventral (Jefferis et al., 2007). Furthermore, in the ant species, *Camponotus obscuripes*, information about alarm pheromones are reported to be more extensively represented in a specific region of the lateral horn than plant odors (Yamagata and Mizunami, 2010).

Generally, the lateral horn is assumed to constitute an integration center that is more closely connected to the motoric system than the calyces—which is shown to be particularly involved in associative memory (Menzel, 2001; Heisenberg, 2003). The segregation of information linked to general odorants vs. pheromones in this particular area, as demonstrated in the current and previous studies, supports the idea of the lateral horn as a region involved in innate behavioral responses. Interestingly, tracing experiments in vertebrates have reported about a separation of particular odor signals in higher brain centers as well; thus, uni-glomerular projection neurons from individual glomeruli in mice were found to display spatially stereotyped and partially overlapping projections in the amygdala (Sosulski et al., 2011). This arrangement, which differs from a more distributed projection pattern in the piriform cortex, has also been suggested to underlie instinctive behavior.

### Representation of pheromone and interspecific signal information in the lateral horn

Heliothine moths are suitable for studying central neuronal mechanisms underlying not only information about multicomponent pheromone blends but also of interspecific signals inhibiting the pheromone attraction (Christensen et al., 1991, 1995; Mustaparta, 1996; Berg et al., 1998, 2005; Vickers et al., 1998; Vickers and Christensen, 2003; Zhao and Berg, 2010). In general, the specificity of pheromone sensory neurons as distinct neuron types conveying information about one component to specific MGC-units of the AL applies to all species studied. Regarding the out-put information from the male-specific glomeruli, most projections neurons identified are reported to maintain, to a certain extent, the specificity of the sensory neurons by responding best to the “key” compound whereas a smaller proportion integrates information about both components (Vickers et al., 1998; Vickers and Christensen, 2003; Zhao and Berg, 2010). As the present study includes MGC-neurons of the first mentioned category only, it was relevant to ask how particular pheromone components are mapped in the lateral horn. In both species used, we found that the major pheromone constituent, being linked to the cumulus, is represented in one distinct region of the lateral horn, namely the medial part of the anterior portion. This means that projection neurons tuned to the major pheromone component—being Z11-16:AL in *H. virescens* and Z9-16:AL in *H. assulta*—were found to target the same area in the lateral horn. Interestingly, this particular region seems to correspond with that innervated by projection neurons tuned to the primary pheromone component, bombykol, in the silk moth, *B. mori* (Seki et al., 2005). The successful staining of one projection neuron tuned to Z11-16:AL in *H. assulta*, enabled mapping of a type tuned to the second pheromone component. In spite of being linked to a relatively small MGC-unit located ventrally in the cumulus, this neuron overlapped completely with the cumulus-neurons in the lateral horn. Thus, the information about the two principal constituents of the pheromone blend which is carried mainly in separate pathways within the mALT, seems to converge in the same region of the lateral horn, i.e., the so-called delta area. This differs from the findings in *B. mori*, where the two pheromone compounds, bombykol and bombykal, are represented in partly different sub-divisions of the lateral horn (Seki et al., 2005). In this species, however, bombykol alone is sufficient for eliciting the male sexual behavior. Taken together, the results indicate that the functional significance of the signal substance—in this case its importance for sexual attraction—is the crucial criterion for the protocerebral target region, and neither the identity of the pheromone substance nor the location or size of the relevant antennal-lobe glomerulus.

The other question of particular interest concerns the mechanism for interspecific interruption of pheromone attraction, triggered by the exposure of pheromone components of a sympatric species. Generally, the presence of specific sensory neurons for pheromone components of a related, neighboring species having an antagonistic effect on attraction, has been demonstrated in many early studies of insect species of different families and genera. In addition, competitive blocking on the receptors has been ruled out by adding the “inhibiting” compounds to the pheromones. Furthermore, a separation of the pheromone and the interspecific signal information has been well documented in several species, including the two heliothine species investigated here, both as concerns input to and output from the MGC (Christensen et al., 1995; Hansson et al., 1995; Berg et al., 1998, 2005; Vickers et al., 1998; Zhao and Berg, 2010). Thus, the next relevant question was whether the separation is kept in the lateral horn. In the present study this issue was particularly resolved by using *H.assulta* having an unusually large population of male-specific receptor neurons and projection neurons tuned to the interspecific signal, Z9-14:AL. The high number of these projection neurons, having arborizations in the dorsal MGC-unit, thus offered the opportunity of exploring their termination area in the lateral horn. As shown from the results presented here, this neuron population displays a characteristic projection pattern in the current region by covering a relatively small area that partly overlaps with the region innervated by the pheromone neurons, but being located slightly more ventrally and posteriorly. Thus, the protocerebral area representing the three MGC-units consists of one relatively large region receiving completely overlapping input from the two types of pheromone neurons and one smaller and partially non-overlapping region targeted by the neurons carrying information about the interspecific signal. Except for pheromone neurons connecting the lateral accessory lobe with the ventral cord, previously found in *M. sexta* and *B. mori* (Kanzaki et al., 1991, 1994), there is little knowledge about higher order neurons in the male-specific pathway of moths. However, the projection pattern described here seems to enable integration of the two pheromone signals in third order neurons. The characteristic representation of the interspecific signal, on the other hand, may permit several alternatives including a continued segregation as well as integration. Taken together, the results obtained from the two species indicate that the male-specific terminals in the lateral horn is to a certain extent spatially organized, not according to the identity of the associated signal substance or the MGC unit they are linked to, but according to their functional significance.

### Projection pattern in the calyces

The mass staining experiments including application of two different fluorescent dyes into particular regions of the same AL, one in the MGC and the other in the OG, uncovered a specific staining pattern in the calyces. Hence, dye application to the MGC revealed an arrangement characterized by overlapping terminals making condensed structures within circular regions of each calyx whereas the dye injected into the OG formed a more distributed projection pattern covering almost the whole structure (Figure 2). These findings are in agreement with previous reports in *M. sexta* (Homberg et al., 1988). Whether the neurons carrying information about the primary pheromone component form a different projection pattern in the calyces from those tuned to the secondary constituent, like in *B. mori* (Namiki et al., 2013), or whether the interspecific information has a unique representation, is impossible to determine from the current data. The reason why the two neuron types carrying information about plant odors and pheromones did not reveal different projection patterns in the AMIRA models may be related to the relatively complex innervation pattern of the individual neurons in the calyces. The main axon of each neuron usually extends 3–5 processes which give off further branches within the wall of the cup-shaped structure. Thus, very small displacements of the reconstructed elements within such a structure will probably disturb the pattern that is there in nature.

### Separation of pheromone signals within the medial ALT

The fact that the information about the two principal pheromone components are kept mainly separated along the mALT via two physiologically distinct neuron categories, as shown here and in several previous studies on heliothine moths (Vickers et al., 1998; Vickers and Christensen, 2003; Zhao and Berg, 2010), justifies a short comment. The purpose of keeping the general pheromone information segregated from the inter-specific signal information at this level seems obvious, based on the opposite behavioral responses induced by the different signals. The meaning of carrying information about the two *pheromone* constituents in distinct second-order neurons, on the other hand, does not automatically seem plain as these signals are responsible for one behavioral response. The separation at the present level, however, may be related to the fact that heliothine moths produce partly similar pheromone components in various ratios, plus their utilization of particular constituents as interspecific signals for preserving the species. Thus, a constituent used as a pheromone component in one species often serves as an interspecific signal in another species. A neural arrangement comprising second-order projection neurons primarily tuned to one distinct signal molecule may have been beneficial for the possibility of evolving new species based on existing systems that can be altered by utilizing the signal molecules in new combinations.

## Conflict of interest statement

The authors declare that the research was conducted in the absence of any commercial or financial relationships that could be construed as a potential conflict of interest.
